# Nanostructured Lipid Carriers (NLC)-Based Topical Formulation of Hesperidin for Effective Treatment of Psoriasis

**DOI:** 10.3390/pharmaceutics17040478

**Published:** 2025-04-07

**Authors:** Anita Rani, Rajwinder Kaur, Afaf Aldahish, Rajalakshimi Vasudevan, Prasanalakshmi Balaji, Chander Parkash Dora, Balakumar Chandrasekaran, Thakur Gurjeet Singh, Rahul Sharma

**Affiliations:** 1 Chitkara College of Pharmacy, Chitkara University, Rajpura 140401, Punjab, India; 2 Department of Pharmacology, College of Pharmacy, King Khalid University, Abha 61421, Saudi Arabia; 3 Department of Computer Science, College of Computer Science, King Khalid University, Abha 61421, Saudi Arabia; 4 Faculty of Pharmacy, Philadelphia University, P.O. Box 1, Amman 19392, Jordan; balakumar@philadelphia.edu.jo; 5 Gateway College of Pharmacy, Sonepat 131001, Haryana, India

**Keywords:** Box–Behnken Design, high-shear homogenization, hesperidin, nanostructured lipid carriers, psoriasis

## Abstract

**Background:** Various routes of drug administration are available for psoriasis treatment. However, there is an urgent need for novel and improved therapeutic options. Hence, our study aimed to develop a nanostructured lipid carrier (NLC) gel of hesperidin (HPD) using a systemic QbD approach for an effective treatment of psoriasis. **Methods**: Initially, HPD-NLC was optimized with independent variables (drug content, amount of liquid lipid, total lipid, and surfactant concentration) using Box–Behnken Design to assess dependent variables (particle size, size distribution, and entrapment efficiency). HPD-NLC was developed using the high-shear homogenization technique. The characteristics of nanoformulation such as particle size, morphology [transmission electron microscopy (TEM) and differential scanning calorimetry (DSC)], crystallinity [powder X-ray diffraction (XRD)], and chemical interactions [Fourier transform infrared spectroscopy (FTIR)], the drug entrapment efficiency (%EE), and the drug release were investigated. Franz-diffusion cell was utilized to perform in vitro diffusion study, and an imiquimod-induced psoriasis model was used for in vivo study. **Results**: The optimized HPD-NLC exhibited a spherical shape with particle size of 125.7 nm, polydispersity index (PDI) of 0.36, and entrapment efficiency of 52.26% *w*/*w*. Further, different techniques validated the reduced crystallinity of the hesperidin. The in vitro diffusion study highlighted the sustained and anomalous diffusion of the drug from NLC gel. In the in vivo study, the HPD-NLC-Gel-treated group displayed normal skin with minimal keratosis, while the drug-loaded gel group exhibited signs of hyperkeratosis and parakeratosis signs. **Conclusions**: HPD-NLC gel showed promising advancement in nanotechnology-based psoriasis treatment and the results of this study open the door for the application of topical HPD-NLC-Gel clinically.

## 1. Introduction

Chronic, immune-mediated skin illness, known as psoriasis, can cause severe morbidity and mortality due to epidermal keratinocyte hyperproliferation and poor differentiation. Effective psoriasis treatment can lead to the restoration of skin to a clinically normal state, characterized by the resolution of epidermal thickness and a reduction of inflammation [1,2]. Approximately eighty percent of psoriasis patients have intense to moderate signs and symptoms, which are treated with topical medications. Those with more advanced disease are typically treated with phototherapy and systemic therapies. It is still recommended to treat psoriasis with topical medication due to its integral advantages, which include less systemic toxicity, enhanced patient compliance, and localized therapeutic impact [3]. The primary limitation of topical therapies is inadequate drug penetration through scaly psoriatic skin. For this reason, the discovery of a novel carrier system for treatment that can deliver a therapeutic amount of drug into the psoriasis-affected tissues is now underway [4].

Hesperidin (HPD) is a flavanone glycoside found naturally present in all citrus fruits. Research has demonstrated that HPD has inhibitory effects on keratinocyte proliferation and exhibits anti-psoriatic properties in murine models with imiquimod-induced psoriasis-like dermatitis [5]. Oral formulations of HPD have not worked well because of its low solubility with limited absorption. The bioavailability of many oral medications often poses challenges due to inadequate solubility, low dissolution rates, and an absence of proportionality in dosages [6]. When patients are obliged to adhere to a frequent dosing schedule for an extended duration of treatment, they typically stop complying with the prescribed therapy. Lipid nanoparticle systems are typically preferred for dermal treatment because they aid in the medication fragment’s operative retention and permeation to the skin and have a greater capacity to entrap drug particles within lipid layers [7,8].

Innovative nanotechnology has been employed to meet the objective of safe and efficacious psoriasis therapy. New topical transporters have been tested to improve skin permeation, including microemulsion, niosomes, nanogel, deformable liposomes, liposomal hydrogel, and solid lipid nanoparticles (SLNs). However, these carriers often face challenges such as low medication encapsulation efficiency, drug instability during storage, and high water content in the formulations. Research on topical and cosmetic treatments utilizing nanostructured lipid carriers (NLC) has gained attention, as these systems effectively address critical problems associated with other nanocarriers. NLC is a second generation of solid lipid nano-compartments stabilized by surfactants, encapsulated in a solid lipid matrix that is spatially incompatible. They enhance the drug loading by preventing the premature release of the encapsulated drug due to its imperfect crystal structure. Furthermore, when applied topically, NLCs improve skin emollience and penetration by providing the skin with deeper hydration [9]. Additionally, NLCs can encapsulate drugs in their molecular state or as amorphous clusters and adhere well to the skin, forming thin films that create an occlusive effect, thereby reducing the transepidermal water loss [10,11]. The current research work aimed to develop an HPD-loaded NLC gel and evaluate its potential in an animal model of psoriasis. A Box–Behnken Design (BBD) was employed to optimize the HPD-NLC formulation. By dissolving the HPD-NLC in the gel base, the HPD-loaded NLC gel was formulated and characterized. The effectiveness of the gel in treating imiquimod-induced psoriasis in Wistar rats was assessed based on histological analysis and the psoriatic area and severity index (PASI) score.

## 2. Materials and Methods

Hesperidin was received as a gift from Harveer Herbal Pharma, Ludhiana, India. Compritol^®^ 888 ATO was obtained from Gattefosse India Private Limited, Mumbai, India. Tween 80, Oleic acid, Hydroxy propyl methyl cellulose (HPMC) K4M, Carbopol 934, acetone, and dichloromethane were purchased from Loba Chemicals Private Limited, Mumbai, India. Imiquad™ cream (Glenmark, Goa, India) was used to induce psoriasis. All the chemicals used in this study were of analytical grade.

### 2.1. Fabrication of Hesperidin-Loaded Nanostructured Lipid Carriers (HPD-NLC)

**Figure 1:**
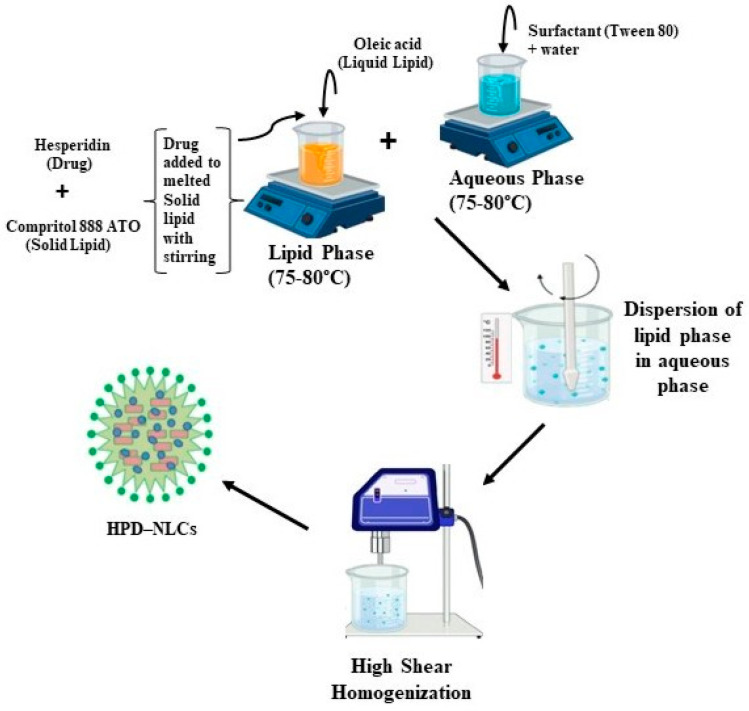
Scheme of the production of HPD–NLC.

The HPD-NLC was formulated using a high-shear homogenization technique [12,13,14] (Figure 1). The formulation process was initiated by preparing the lipid and aqueous phases separately. The lipid phase consisted of oleic acid as the liquid lipid, Compritol^®^ 888 ATO as the solid lipid, and the drug, while the aqueous phase contained a solution of Tween 80 solution as the surfactant. Both phases were heated individually heated to a temperature range of 75–80 °C for about 10 min. The aqueous phase was gradually added into the lipid phase while continuously mixing using a high-shear homogenizer set at an optimal speed. This process was continued for an additional 10 min to ensure the successful fabrication of drug-loaded NLC.

### 2.2. Statistical Experimental Design

A response surface design known as BBD does not include embedded factorial or fractional factorial designs. Compared to central composite designs (CCD) with an equivalent total number of trials, BBD offers a more cost-effective approach. The Design-Expert software (Stat-Ease 360 trial version) was used for statistical analysis and experimental design. This study used a BBD that combined three levels with four parameters. To evaluate the link between the independent and dependent variables, BBD was used for the optimization [15,16]. The quantity of drug (A), total lipids (B), liquid-lipid (C), and surfactant concentration (D) were the four independent variables, while particle size (nm) (Y1), polydispersity index (PDI) (Y2), and entrapment efficiency (EE) (Y3) were the three dependent (response) variables considered during the BBD process to produce the formulations for HPD-NLCs (Table 1). A total of thirty batches were created and evaluated to carry out this formulation.

### 2.3. Lyophilization

With minor modifications, the prepared formulations were freeze-dried using a Lyophilizer (FD-3, Biotech Systems, New Delhi, India) and a previously created stepwise freeze-drying cycle [17]. The freeze-drying cycle consists of initial eight hours of freezing at −60 °C, followed by forty-two hours of primary (1°) drying from −60 °C to 20 °C, and two hours of secondary (2°) drying at 25 °C. During all stages, a constant pressure of 200 mTorr was maintained. At a 5% *w*/*v* concentration, an original selection of various cryoprotectants, including mannitol, dextrose, trehalose, and inulin, was conducted. The appearance of the lyophilized cake, along with the re-dispersibility index and re-dispersibility time of the freeze-dried formulations were evaluated.

### 2.4. Characterization of HPD-NLC

#### 2.4.1. Particle Size and PDI of Optimized HPD-NLC

The nanosize of the HPD-NLC preparation was determined in the dynamic analysis outcomes. Using Zetasizer (ZSU3100, Malvern Panalytical, UK) and dynamic light scattering, the particle size and PDI of HPD-NLC were determined at room temperature and a fixed angle of 90°. HPLC-grade water (Sigma-Aldrich Chemicals Private Limited, Bangalore, India) was added (10 times dilution) to the NLC suspension to determine the particle size. Upon completion of all measurements (triplicate), their average size was determined [18].

#### 2.4.2. EE of Optimized HPD-NLC

The ultra-filtration technique was used to determine the EE of HPD-NLC. The amount of medication entrapped was calculated based on the free drug concentration in NLC [18]. A precisely weighed quantity of HPD-NLC was ultra-filtered using a filter membrane (Durapore^®^, PVDF 0.45 µm, Merck Life Science Private Limited, Mumbai, India). After the ultra-filtrate dilution with a phosphate buffer solution of pH 6.8, the drug content in the ultra-filtrate was determined using a double-beam UV-visible spectrophotometer (Systronics, Mumbai, India) at 285 nm. The following formula was used to calculate the %EE of the formulations.

%EE = Total amount of drug − Total amount of un-entrapped drug/Total amount of drug × 100

#### 2.4.3. High-Resolution Transmission Electron Microscopy (HRTEM) of Optimized HPD-NLC

The NLCs’ morphology was studied using HRTEM (TEM FEI Tecnai G2 F20, Amsterdam, The Netherlands). A drop of NLC suspension (100 mg/mL) was placed on a copper grid that had carbon covering on it. It was then stained with phosphotungstic acid (2% *w*/*v*) and allowed to dry. Images were captured at different magnifications using an accelerating voltage of 200 kV. The size distribution was ascertained by a comprehensive software analysis of an HRTEM image [19].

#### 2.4.4. Differential Scanning Calorimetry (DSC) of Optimized HPD-NLC

DSC analysis aimed to observe any thermal changes resulting from the incorporation of the drug into the nanocarriers, providing insights into the formulation’s stability and compatibility. The thermal behaviour of the optimized formulation, including HPD, Compritol^®^ 888 ATO, oleic acid, and HPD-NLC was analyzed using a DSC 4000 (PerkinElmer GmbH, Rodgau, Germany). Before conducting the analysis, Indium was used to calibrate the temperature range and cell constant measurements. The analysis was conducted over a temperature range of 25–200 °C at a heating rate of 10 °C/min with nitrogen purging (100 mL/min). A reference empty pan was utilized for analysis when samples weighing 2–8 mg were placed into an aluminum pan [19].

#### 2.4.5. X-ray Diffraction (XRD) Analysis of Optimized HPD-NLC

XRD analysis was used to identify the changes in the polymorphic nature of the physical mixture, surfactant, and pure HPD. An X-ray diffractometer (X’PERT-PRO, Malvern Panalytical, Malvern, UK) was used to analyze the NLCs. The X-ray source was the Cu Kα tube, which has a wavelength of 1.5406 Å operating at 40 kV and 40 mA. The samples were scanned between 4 °C and 40 °C (2θ), at 0.1 (2θ)/min [17].

#### 2.4.6. Fourier Transform Infrared (FTIR) Spectroscopy of Optimized HPD-NLC

To validate the absence of any chemical interactions between the constituents in the formulations, an FTIR analysis was carried out. After fully mixing freeze-dried drug-loaded HPD-NLCs with KBr in a 1:100 ratio, an IR-transparent matrix pellet was created. An FTIR spectrometer (Shimadzu Analytical Private Limited, Mumbai, India) was then used to scan the pellet over a wave range of 4000–400 cm^−1^ to record the spectra [20].

### 2.5. In Vitro Evaluation of Optimized HPD-NLC

#### 2.5.1. In Vitro Drug Diffusion Study

It is well established that several variables including the composition and structure of the NLC can affect the diffusion of drugs from nanocarriers. Thus, the in vitro discharge of HPD from NLCs was investigated. The dialysis bag diffusion technique was used to investigate in vitro drug release in phosphate buffer pH 6.8. Twelve hours before use, the dialysis bag membrane with the molecular weight cut off at 12–14 kDa was soaked in phosphate buffer saline (PBS) solution. The dialysis bag was magnetically agitated in a beaker with 250 mL of dissolving medium (PBS pH 6.8) at 100 rpm and 37 ± 2 °C. 2 mL of the NLCs dispersion was added to the dialysis bag, which was then securely closed from one end. Later, it was once again magnetically stirred in a beaker with 200 mL of PBS at 50 rpm and 37 ± 2 °C. To maintain sink conditions, 5 mL of the dissolution medium was taken out and replaced with the new buffer at periodic times of 0, 1, 2, 3, 4, 5, 6, 7, 8, 9, 10, and 24 h. The filtrated solution was appropriately diluted before being examined at 285 nm using a double-beam UV-visible spectrophotometer (Systronics, Mumbai, India) [20,21]. All analyses were performed in triplicate.

#### 2.5.2. Release Kinetics

Various kinetic models, such as zero-order, first-order, Higuchi, and Korsmeyer–Peppas, have been used to explain the process of the release of drugs from HPD-NLC. MS Excel 2016 software is used and the release values have been fitted to the zero-order, first-order, Higuchi, and Korsmeyer–Peppas models, which show the total quantity of the drug released over time, to investigate the drug release kinetics. When the release mechanism is unknown or when many release phenomena are involved, these model-dependent techniques are used to analyze the in vitro release behavior of the drug (HPD) from its formulation system. The correlation coefficient (R2) was selected to establish the accuracy and predictive ability of these models. To characterize the in vitro release of HPD from the formulation, the kinetic model was believed to be the most appropriate with the greatest correlation coefficient (R^2^) [22].

#### 2.5.3. Stability Study of Optimized HPD-NLCs

Long-term and accelerated stability studies of optimized HPD-NLCs were carried out in compliance with ICH (International Council on Harmonisation) Q1A (R2) guidelines at 25 ± 2 °C/60 ± 5% RH and 40 ± 2 °C/75 ± 5% RH, respectively. The change in % EE of optimized HPD-NLCs was measured at predetermined time intervals such as day 0, 1, 3, 6, 9, and 12 months after storage [23].

### 2.6. Preparation of Optimized HPD-NLC Loaded Topical Gel (HPD-NLC-Gel)

Topical gels filled with HPD-NLC were prepared using a dispersion process with HPMC K4M and Carbopol 934. Each component was precisely weighed and taken (Table 2). Both polymers were dissolved in water and left for four hours to allow the polymer to properly expand. To prevent the entrapment of air bubbles, HPD-NLC (equal to 7.5% *w*/*w*) was added to the gel while being continuously mixed in a single direction. After thoroughly mixing the gel mixture, any air bubbles were eliminated using a Sonicator (JP-030S, Raj Analytical Solutions Private Limited, Punjab, India). To enhance the cross-linking between polymers and produce a transparent gel, triethanolamine was then added to a polymeric mixture of Carbopol 934 and HPMC K4M. The viscosity of the gel was balanced by adding glycerol [24].

### 2.7. Characterization of Optimized HPD-NLC-Gel

#### 2.7.1. Determination of the Physical Appearance of HPD-NLC-Gel

The physical appearance of the HPD-NLC-Gel was visually examined for its consistency, texture, homogeneity, and color uniformity. A small amount of the gel formulation was rubbed between the thumb and index finger to test its homogeneity and gel texture (visualization method). The consistency of the gel, as well as the presence or absence of gritty or undissolved components, were taken into consideration to determine whether or not the gel was uniform and smooth [18].

#### 2.7.2. Measurement of pH Value

The pH was evaluated by taking the HPD-NLC-Gel (250 mg) and adding it to distilled water (50 mL). The pH was determined using a digital pH meter (Deluxe pH meter-101 model, Electronics India, Panchkula, India). Maintaining an appropriate pH range is essential, as changes may impact the skin’s protective functions. Therefore, to prevent changes in the pH of the skin, topical formulations should have a pH range between 6 and 7 [12,23].

#### 2.7.3. Measurement of Viscosity

The Brookefield DV-II Pro Viscometer (Brookfield Engineering Laboratories Inc, Middleboro, MA, USA) at 25 ± 1 °C with spindle number S-64 at different shear rates was used to determine the viscosity of HPD-NLC-Gel [18,23].

#### 2.7.4. Measurement of Spreadability

The horizontal glass plate method was used to assess the spreadability of the formulations. After placing the HPD-NLC gel (1 g) on a pre-marked glass plate (1 cm in diameter), the second glass plate was left over for 5 min [25]. The increase in diameter caused by the gel spreading was computed using the following equation:

% Spread area = A2/A1 × 100

where A1 = initial area (cm^2^) and A2 = final area (cm^2^) after spreading [12,17,24].

#### 2.7.5. Drug Content

One g of the gel, precisely weighed, was added to a 100 mL volumetric flask containing 20 mL of phosphate buffer (pH 5.5). After 30 min of shaking, the volumetric flask was filled to 100 mL with a phosphate buffer pH 5.5. After suitable dilution, the sample was analyzed using a UV-visible spectrophotometer at 285 nm [23]. The following equation was used to calculate the percentage drug content:

% Drug Content = Actual drug content/Total drug amount taken × 100

#### 2.7.6. In Vitro Drug Diffusion Studies of HPD-NLC-Gel

Franz diffusion cells were used for the in vitro diffusion study of HPD formulations. The sections of the Franz diffusion cell were separated by a cellophane membrane [26]. A mixture of 5 mL of phosphate buffer (pH 6.8) and methanol (80:20) combination was added to the acceptor compartment as release media, which was agitated at 50 rpm (receptor medium). A constant temperature of 37 ± 0.5 °C was maintained. One side of the dialysis membrane-110 LA 395-1MT (2 × 2 cm, molecular weight cut off: 1200–14,000 Dalton) procured from HiMedia Laboratories Private Limited, Mumbai, India (donor compartment) was covered with 1 g of HPD-Gel, HPD-NLC-Gel, and physical mixture gel (PM-Gel). Then, 2 mL of samples were taken out of the receptor media at various intervals (0, 1, 2, 3, 4, 5, 6, 8, 10, 12, and 24 h), filtered, and their drug concentration was measured using a double-beam UV-visible spectrophotometer (Systronics, Mumbai, India) at 285 nm. All the studies were conducted in triplicate [23,25,26,27].

#### 2.7.7. Stability Study of HPD-NLC-Gel

At specified intervals of 3, 6, 9, and 12 months, the pH and viscosity changes in the HPD-NLC-Gel were assessed at 5 ± 3 °C, 25 ± 2 °C, and 60 ± 5% RH, and 40 ± 2 °C and 75 ± 5% RH according to ICH recommendations. The best formulation was kept in an aluminum foil-sealed container at room temperature [23,27].

### 2.8. In Vivo Study of Optimized HPD-NLC-Gel

#### 2.8.1. Animals and Ethics Statement

Wistar rats (7–8 weeks old) of either sex, weighing between 180 to 220 g were used as per experimental protocols (IAEC/CCP/23/02/PR-13) after consent from the Institutional Animal Ethical Committee (IAEC) and Chitkara College of Pharmacy (CCP), Chitkara University, Rajpura, Punjab, India. The animals were housed under standard environmental conditions (25 ± 2 °C), as per Organization for Economic Cooperation and Development (OECD) guidelines. They were kept in an environment with a twelve-hour photocycle at typical temperatures (22 °C to 27 °C) and relative humidity levels (65%). All experiments were conducted following the instructions published by the Committee for Control and Supervision of Experiments on Animals (CCSEA) under the provisions of the Prevention of Cruelty to Animals Act, 1960, governed by the Ministry of Environment, Forest and Climate Change, Government of India. Each animal was fed a standard pellet diet and had unrestricted access to water. Animals were acclimatized for 1 week before the experiments and drugs were given through the topical route [26,28,29].

#### 2.8.2. In Vivo Antipsoriatic Activity

Five groups (n = 6) were randomly selected from the animals. Group I was a Normal (disease-free negative control), Group II was a Disease (disease-infected positive control), Group III received a Placebo Gel (blank gel formulation), Group IV was given an HPD-gel formulation, Group V was administered with a Standard (Betagel^®^: Betamethasone 0.05% *w*/*w*) (Micro Labs, Bangalore, India), and Group VI was treated with HPD-NLC-Gel formulation (which is equivalent to 0.045% *w*/*w* of HPD). In all groups except Group I, psoriasis was induced by applying Imiquad^TM^ cream (Imiquimod 5% *w*/*w*) to a shaved back. Each rat received a topical dose of 62.5 mg of imiquimod cream for 10 days, which was equivalent to 3.125 mg of imiquimod each day. Treatment with different formulations was initiated and continued for the next seven days. The antipsoriatic activity potential of the formulations was evaluated using PASI score and histopathological examination [28,29].

#### 2.8.3. PASI Score

The severity of psoriasis was evaluated based on three symptoms of erythema, scaling, and thickening. The PASI score was then separately categorized as 0—none, 1—faint, 2—moderate, 3—marked, and 4—extremely marked. The extent of psoriasis was determined by the sum of the inflammation, edema, and scaling scores.

#### 2.8.4. Histopathological Examination

Animals were sacrificed twenty-four hours after treatment ended, and samples of the afflicted skin were collected and preserved in 10% formalin. For light microscopic analysis, samples were cut transversely and embedded in paraffin. Samples were examined under a light microscope (100OLYMP, Olympus, Tokyo, Japan) after being stained with hematoxylin and eosin (H&E). For each slide, a minimum of five to six fields were analyzed to ensure proper interpretation. From each group, a minimum of twenty-five randomly chosen tissue slices were examined for histopathological alterations such as hyperkeratosis, orthokeratosis, and parakeratosis [28,29].

## 3. Results

### 3.1. Optimization of HPD-NLC

**Figure 2:**
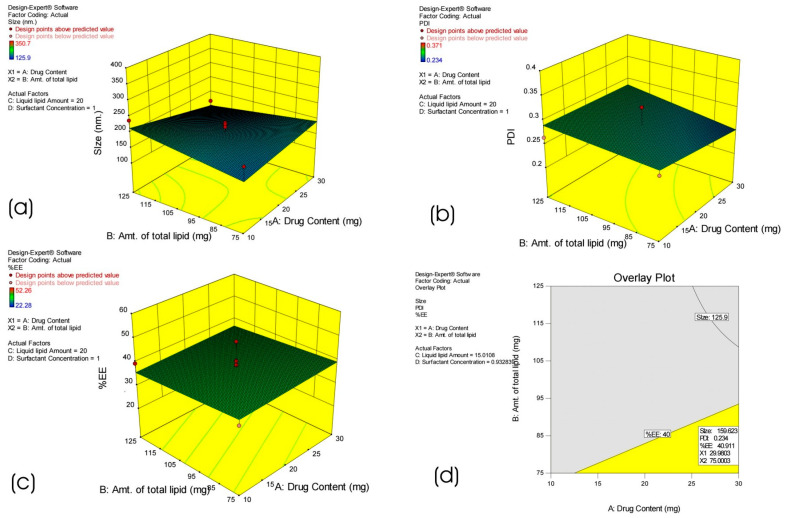
Response surface plot illustrating the impact of independent variables on dependent variables ((**a**) top-left) particle size, ((**b**) top-right) PDI, ((**c**) bottom-left) % EE, and ((**d**) bottom-right) Plot overlay.

In this research, thirty batches of NLCs were formulated by BBD using a combination of four factors at three levels, and design analysis was executed to develop a polynomial equation [30]. The dependent variables such as particle size, PDI, and EE, are highly impacted by the chosen independent variables, including the amount of liquid lipid, total lipid, surfactant concentration, and drug content (Table 2). Response surface analyses, mostly based on the model polynomial functions, are shown in three-dimensional model graphs to show how pre-determined factors affect the response variables (Figure 2a–c). While maintaining the third component at a constant level, the response surface plots were also utilized to examine the effects of two independent variables on the responses or dependent variables. The qualitative impact of all the variables on every outcome parameter could be seen when these plots were closely examined [17,31].

For different factor level combinations, the particle size ranges from 350.7 nm (formulation 8) to 125.9 nm (formulation 26) (Table 2). The effect of liquid lipid (C) and surfactant amount (D) on particle size is explained by the quadratic equation below, with the quantities of drug content (A) and total lipid (B) kept at constant levels:

Size = +179.71 + 8.11A + 2.29B + 28.50C − 9.96D − 23.42AB − 0.55AC + 16.61AD + 3.92BC − 0.10BD − 61.23CD

A positive value before a factor in the regression equation indicates that the response increases with the factor and vice versa. The value of the coefficient makes it clear that liquid lipid content (C) is the most critical factor influencing the variance in particle size. The impact of changing the amount of drug content (A) and total lipid content (B) on particle size was examined in Figure 2a. For the different factor level combinations listed in Table 2, the PDI ranged from 0.234 (formulation 9) to 0.365 (formulation 19). When the amount of liquid lipid and surfactant concentration was held constant, the impact of changing the drug content and total lipid content on the PDI was examined in Figure 2b.

The impact of the surfactant’s quantity on PDI is described by the equation given below:

PDI = +0.28 + 0.006983A + 0.012B + 0.023C − 0.009556D + 0.005529AB + 0.007500AC + 0.032AD + 0.014BC − 0.006BD − 0.039CD

For different factor level combinations, entrapment efficiency changed from 22.28% (formulation 4) to 52.26% (formulation 23), as shown in Table 2. When the amount of liquid lipid and surfactant concentration remained constant, the impact of different drug contents and total lipid content on the entrapment efficiency was examined, as seen in Figure 2c. The surfactant amount (D) also significantly and favorably impacts entrapment efficiency. Entrapment efficiency was considerably enhanced by raising the amount of emulsifier (Figure 2c). The impact of drug content on %EE is described by the equation given below:

%EE = +37.56 + 0.52A − 1.23B − 2.60C + 7.47D

The optimum formulation was built using the desirability function in Design-Expert, aiming for maximum entrapment efficiency, particle size (125–200 nm), and PDI (0.23–0.28). Three new batches of NLC were prepared according to the design space shown in the overlay plot (Figure 2d) to validate the optimization process. The experimental values were within the range of the predicted values within the 5% prediction error, validating the BBD for the optimization of HPD-NLC.

### 3.2. Morphological Characterization of HPD-NLC

#### 3.2.1. HRTEM

**Figure 3:**
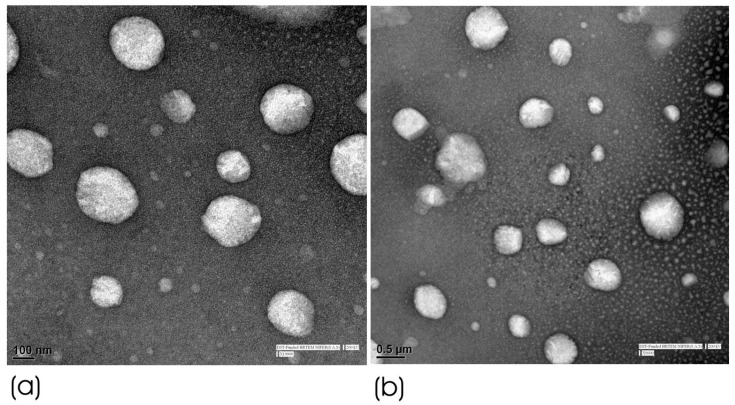
HRTEM of optimized HPD-NLC at different magnifications of (**a**) ×19,000 and (**b**) ×9600.

Figure 3 presents the HRTEM image of HPD-NLC particles, which showed spherical morphologies through a visual inspection of the spherical nanostructures present in the formulation at different magnifications (×19,000 and ×9600).

#### 3.2.2. XRD of Optimized HPD-NLC

**Figure 4:**
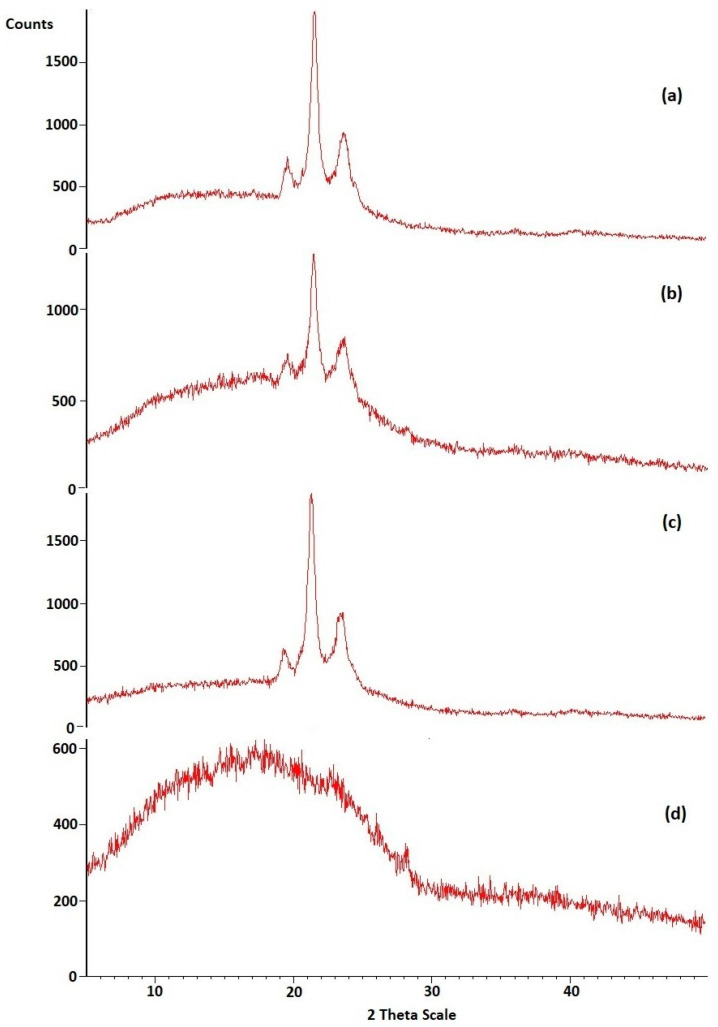
Powder X-ray diffraction patterns of (**a**) pure HPD, (**b**) Compritol^®^ 888 ATO, (**c**) Physical mixture, and (**d**) HPD-NLC.

The X-ray pattern of the pure drug had the highest intensity, and characteristic peaks showed that the drug exists in a crystalline form (Figure 4a), where X-ray diffraction pattern of pure drug illustrated strong specific peaks at 2θ equal to 19.55, 21.5, 23.63, 35.98, and 40.22°, which indicates its high crystal structure and validated the previous work [32]. The X-ray diffractogram of the physical mixture indicated the peaks of lipids and drug thereby confirming their compatibility (Figure 4a–d). The absence of noticeable, crisp patterns in HPD-NLC encouraged the transformation of the drug into an amorphous state. The powder X-ray diffraction patterns of pure HPD, Compritol^®^ 888 ATO, physical mixture, and HPD-NLC are depicted in Figure 4a–d. Moreover, X-ray diffraction peaks are collected in Table 3, Table 4, Table 5 and Table 6.

#### 3.2.3. Fourier Transform Infrared (FTIR) Study

**Figure 5:**
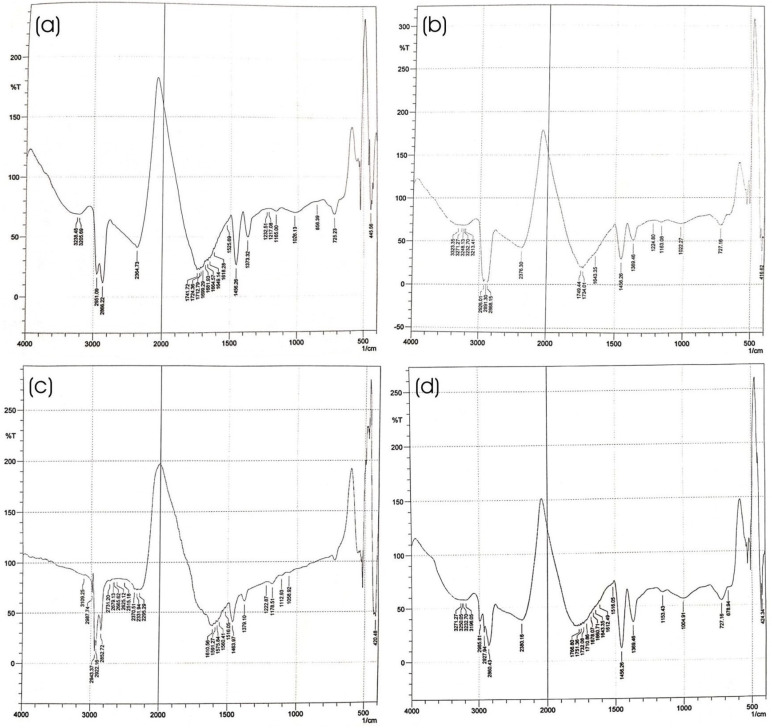
FTIR images of (**a**) HPD, (**b**) Compritol^®^ 888 ATO, (**c**) Physical mixture, and (**d**) HPD-NLC.

Figure 5 presents FTIR images of pure HPD, Compritol^®^ 888 ATO, Physical mixture, and HPD-NLCs. HPD showed characteristic peaks at 1649, 2866, 1026, 2951, and 1456 cm^1^ corresponding to C=O, C–H, C–O, –OH, and C=C stretching frequency, respectively. Compritol^®^ 888 ATO exhibited characteristic peaks at 1749, 2868, 2926, and 1022 cm^−1^, equivalent to C=O (s), C–H (s), C–H (s), and C–O (s), respectively. The characteristic absorption peaks of HPD and Compritol^®^ 888 ATO remained integral in the physical mixture, which revealed compatibility between the drug and lipid. FTIR spectra of HPD-NLC illustrated the presence of prominent absorption peaks of HPD that implied that HPD was successfully incorporated into the lipid matrix and no chemical interactions were observed (Figure 5a–d).

#### 3.2.4. DSC Analysis

**Figure 6:**
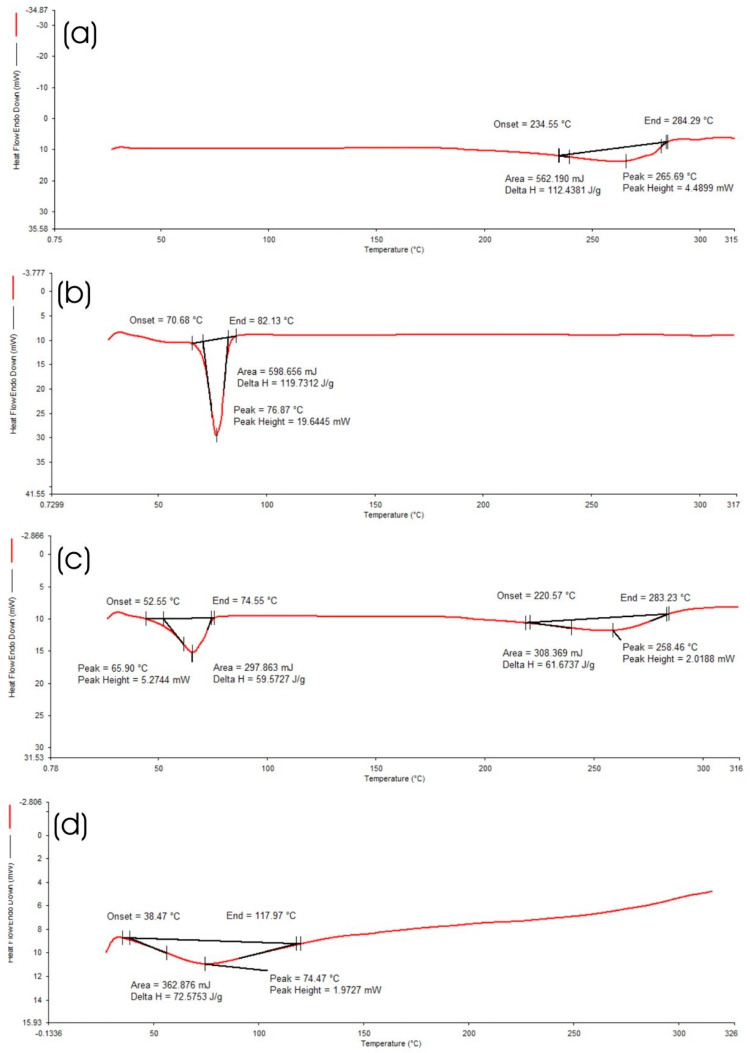
DSC images of (**a**) HPD, (**b**) Compritol^®^ 888 ATO, (**c**) Physical Mixture, and (**d**) HPD-NLCs.

An overview of HPD, Compritol^®^ 888 ATO, and HPD-NLC melting processes is shown in Figure 6. The DSC thermograms for HPD showed a characteristic endothermic peak at 265 °C, corresponding to the drug’s melting point (T_m_) indicating that it is largely crystalline (Figure 6a). Compritol^®^ 888 ATO’s DSC thermogram showed a distinctive melting endotherm at 76 °C (Figure 6b) suggesting that it is a crystalline substance (Figure 6b). The characteristic endothermic peaks associated with drugs and lipids persisted noticeably in physical combinations, which demonstrated the stability of both drug and lipids (Figure 6c). HPD-NLC did not exhibit any distinctive HPD endothermic melting peak, suggesting that the drug exists as an amorphous state within the HPD-NLC (Figure 6d).

#### 3.2.5. Particle Size and PDI

Particle size was significantly influenced by the amount of lipids used. A slight increase in solid lipid content significantly increased the particle size, whereas the particle size decreased as the amount of liquid lipid increased. Better transportation of drugs through the skin largely depends on the presence of smaller particle sizes [33]. Generally, the NLC formulation has a restricted size range (10–1000 nm) distribution, with PDI ≤ 0.5. The particles in the 50–300 nm size range show faster release, while particles larger than 300 nm offer sustained drug delivery. HPD-NLCs exhibited a characteristic spherical morphology with a particle size of 125.7 nm and 0.36 PDI.

### 3.3. In Vitro Diffusion Study of Optimized HPD-NLC

**Figure 7:**
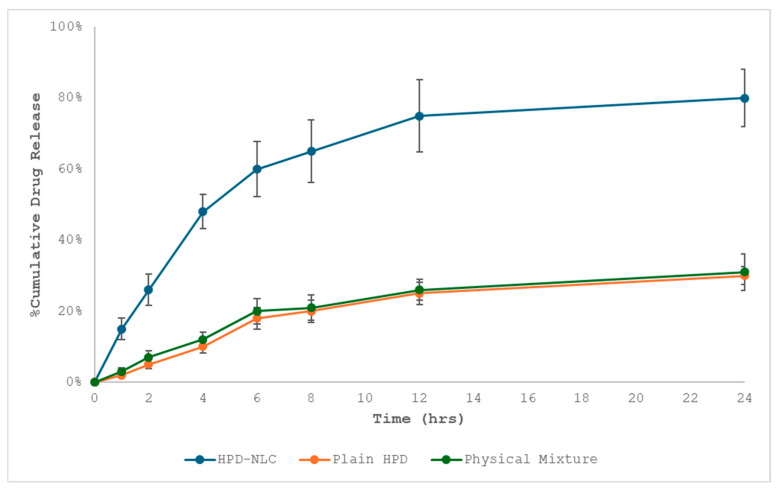
In vitro drug release pattern from optimized HPD-NLC, HPD, and physical mixture.

A dialysis membrane with a molecular weight of 12,000–14,000 Da and a surface area of 2.54 cm² was placed across the cell segments of a Franz diffusion cell after being soaked in double-distilled water [34]. To decrease the stalled surfaces, the receiving segment was charged with 1% sodium lauryl sulphate (SLS) in phosphate buffer of pH 6.8 (20 mL) as the receptor media and agitated at 200 rpm using a magnetic bar in the acceptor medium. For in vitro dissolution studies, 1 mL aqueous dispersion of HPD, HPD-NLC, and physical mixture was placed in the donor compartment. To determine dissolution efficiency, a sample of 2 mL was collected at every predetermined time point (0.5, 1, 2, 4, 6, 8, 10, 12, and 24 h), and HPD concentration was analyzed spectrophotometrically at 285 nm. Approximately 27.61% and 28.48% of the drug was released from HPD and physical mixture, respectively, within twelve hours. On the other hand, HPD-NLC demonstrated a biphasic release pattern, with a two-hour “burst release” of roughly 32.45% partially bound HPD close to the outer layer of lipid particles followed by a four-hour release of 48.85% drug. Later, the highest release rate of 81.34% over twenty-four hours was attained, indicating uniform trapping and even drug dispersion in the lipid matrix. This was the result of sustained drug release caused by diffusion from the lipid matrix (Figure 7).

### 3.4. Release Kinetics of Optimized HPD-NLC

**Figure 8:**
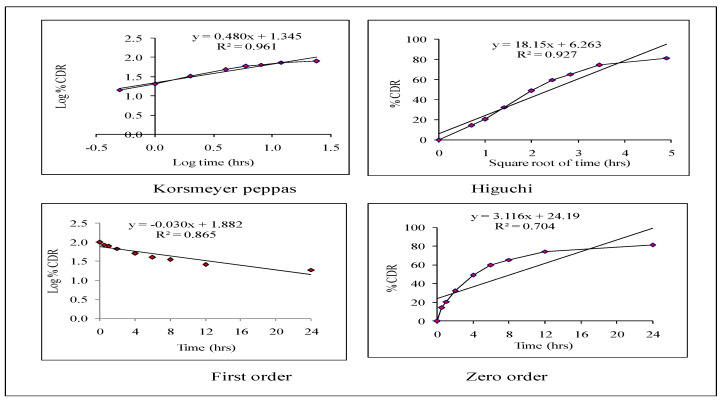
Different in vitro drug release kinetic models for optimized HPD-NLC.

Korsmeyer–Peppas, Higuchi, zero-order, and first-order kinetic models are among the specific models that have been used to explain the mechanism of release of drugs from HPD-NLC. Fickian diffusion, anomalous diffusion, case II transport, and supercase II transport are all covered by the release exponent values of <0.45, 0.46, 0.89, and >0.89, respectively [35,36]. Higuchi, first-order, zero-order, and Korsmeyer–Peppas models were found to have association values of 0.927, 0.865, 0.704, and 0.961, respectively (Figure 8). According to the findings, the Korsmeyer–Peppas model provided the greatest fit for the medication release kinetics from HPD-NLC.

### 3.5. Stability Study of Optimized HPD-NLC

**Figure 9:**
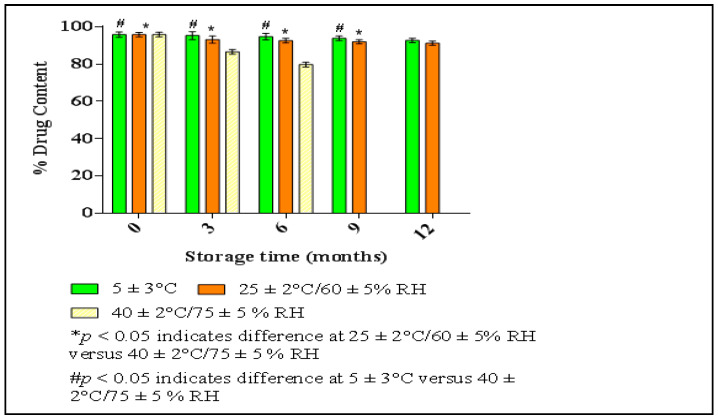
Percentage change in drug content of optimized HPD-NLC against storage time.

The percentage change in drug content at predefined intervals of 3, 6, 9, and 12 months was used to characterize optimized HPD-NLC (Figure 9). When samples were stored at 40 ± 2 °C and 75 ± 5% RH, the percentage of drug content changed significantly compared to samples stored at 25 ± 2 °C and 60 ± 5% RH, and 5 ± 3 °C (*p* > 0.05).

### 3.6. Formulation of Optimized HPD-NLC-Gel

An optimized topical gel filled with HPD-NLC was created using a dispersion process, with each component precisely weighed as the amounts indicated in Table 7. This optimized HPD-NLC-Gel was further characterized and evaluated by in vitro and in vivo studies.

### 3.7. Physical Characterization of Optimized HPD-NLC-Gel

#### 3.7.1. Determination of the Physical Appearance

The optimized HPD-NLC-Gel was found to be cosmetically appealing, smooth to the touch, non-gritty, and homogenous upon visual examination. 

#### 3.7.2. Spreadability

The spreadability of the HPD-NLC-Gel was assessed by measuring the spreading diameter of 1 g of sample between two horizontal glass plates with the upper plate subjected to a normal weight of 25 g for one minute. It took 16 ± 2 s for the top glass to move over the bottom glass. The spreadability results of the HPD-NLC-Gel indicated that the formulated gel spread with an influence of a small quantity of shear [35].

#### 3.7.3. pH Value

The pH of the HPD-NLC-Gel was found to be 6.5 ± 0.3, which suggests less skin irritation when applying the specially designed HPD-NLC loaded Gel.

#### 3.7.4. Viscosity

The prepared HPD-NLC-Gel was studied for its rheological behavior at 25 ± 1 °C using Brookefield viscometer, and the viscosity of the formulated gel was found to be 3546 ± 47 centipoises, which complied with the theoretical values [36].

#### 3.7.5. Stability Study of HPD-NLC-Gel

The pH and viscosity changes of the HPD-NLC-loaded topical gel were evaluated at specified intervals of 3, 6, 9, and 12 months. Gels maintained at 40 ± 2 °C and 75 ± 5% RH to those placed at 25 ± 2 °C, 60 ± 5% RH, and 5 ± 3 °C, a significant reduction in viscosity was observed (*p* > 0.05). The HPD-NLC Gel formulation was found to be stable while stored at 25 ± 2 °C, 60 ± 5% RH, and 5 ± 3 °C. However, no significant difference in pH was observed at any of the studied storage conditions (Table 8).

### 3.8. In Vitro Drug Release Study of HPD-NLC-Gel

**Figure 10:**
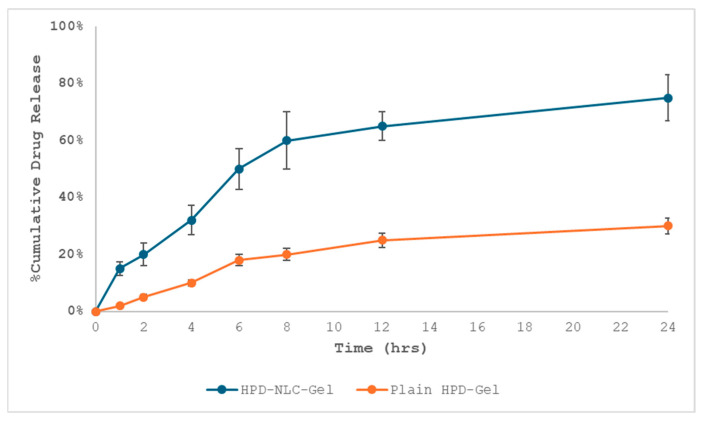
In vitro drug release pattern from HPD-NLC-Gel versus HPD-Gel.

The cellophane membrane was mounted between the cell compartments of the Franz diffusion cell [35,36,37,38]. The acceptor compartment was filled with phosphate buffer, pH 6.8 as receptor medium, and stirred by the magnetic bar to minimize stagnant layers. One g of HPD-Gel, HPD-NLC-Gel, and Physical mixture gel (PM-Gel) was placed in the donor compartment. Then, 2 mL of samples were withdrawn at different time intervals and were analyzed by UV-vis spectrophotometer at 243 nm. While HPD-NLC showed a biphasic release pattern with an initial “burst release” of roughly 25.29% loosely bound HPD on or near the surface of lipid particles during the first two hours, followed by the release of 33.74% drug within the next four hours, approximately 25.52% of the drug was released from HPD within 12 h. The drug was then released continuously from the lipid matrix due to diffusion, reaching a maximum release of 76.35% over twenty-four hours. This suggested that the drug was evenly entrapped and dispersed throughout the lipid matrix (Figure 10).

### 3.9. Release Kinetics from HPD-NLC-Gel

**Figure 11:**
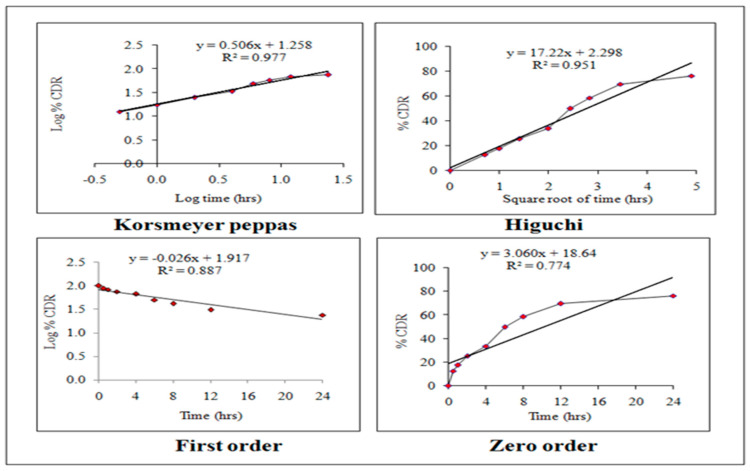
Different in vitro drug release kinetic models of HPD-NLC-Gel.

To explain the mechanism of drug release from HPD-NLC-Gel, kinetic models (Korsmeyer–Peppas, Higuchi, zero-order, and first-order models) were used. The release exponent values for Fickian diffusion, anomalous diffusion, case II transport, and supercase II transport are <0.45, 0.46–0.88, 0.89, and >0.89, respectively [37,38]. According to Figure 11, the correlation coefficients for the zero-order, first-order, Higuchi, and Korsmeyer–Peppas models were 0.774, 0.887, 0.951, and 0.977, respectively.

### 3.10. In Vivo Antipsoriatic Activity of HPD-NLC-Gel

**Figure 12:**
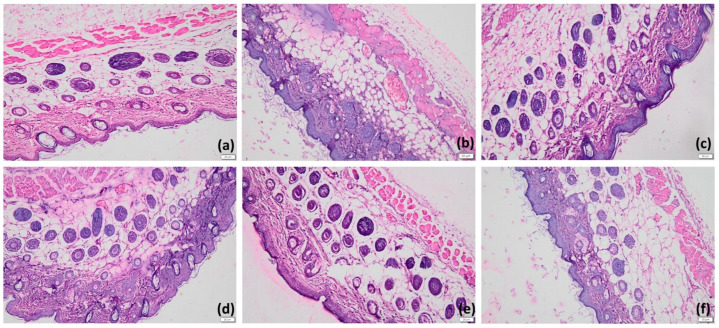
Histopathology of skin samples (20×, H&E staining). Normal (**a**), Disease (**b**), Placebo gel (**c**), HPD-Gel (**d**), Standard treatment (**e**), and HPD-NLC-Gel (**f**).

Several studies have shown that imiquimod stimulates immune cells via toll-like receptors, triggering psoriasis-like inflammation [39,40]. In an experiment using Wistar rats, typical psoriasis symptoms—such as redness (erythema), scaling, and skin thickening—appeared after ten days of applying imiquimod on their shaved backs. On the eleventh day, treatment has been initiated. The psoriasis severity (measured by the PASI score) was initially 2.3. By the end of the treatment period, the scores improved significantly to 0.3 for the HPD-NLC-Gel group, 1 for the standard treatment group, 1.3 for the HPD-Gel group, and 1.7 for the Placebo gel group. In contrast, the disease control group showed no improvement (Table 9). Interestingly, animals treated with the HPD-NLC-Gel also exhibited hair regrowth, hinting at the recovery of hair follicles and the regeneration of healthy skin. Histopathological images from different treatment groups are presented in Figure 12. In this examination, the normal control group exhibited intact, healthy skin without any signs of keratosis, while the placebo gel group showed mild hyperkeratosis and orthokeratosis, likely due to slight skin sensitivity. The disease control group, however, displayed severe hyperkeratosis and parakeratosis, confirming the successful induction of psoriasis-like symptoms. Groups were treated with the HPD-Gel formulation, and the standard treatment showed minimal signs of hyperkeratosis and parakeratosis. Notably, the HPD-NLC-Gel group demonstrated nearly normal skin with mild keratosis, attributable to enhanced drug penetration and sustained release.

## 4. Discussion

This study aimed to formulate and optimize the topical delivery system for hesperidin-loaded nanostructured lipid carrier gel as a novel approach for treating of psoriasis. The optimization process was guided by BBD and had a significant influence of lipid surfactant ratio on output variables resulting in a stable nanocarrier with optimized entrapment efficiency. The selection of Compritol^®^ 888 ATO (glyceryl dibehenate) as a solid lipid and oleic acid as a liquid lipid was advantageous in the formulation of HPD-NLC, as both are useful to enhance the permeability of the drug [41]. Moreover, Tween 80 was used as a surfactant in the formulation to increase the solubility of a poorly soluble drug, increase the penetration of the drug, and improve the stability of oil droplets by creating a mechanical barrier around these oily carriers [42]. DSC and XRD analyses confirmed the conversion of hesperidin to an amorphous state, thus affirming the encapsulation and solubility enhancement. These findings are consistent with previously reported studies on lipid-based nanocarriers, as they have been shown to improve drug solubility and permeability owing to molecular dispersion of drug in lipid matrix [43].

HPD-NLC gel was formulated, where Carbopol (a crosslinked copolymer of acrylic acid) is used as a gelling agent, which is easy to disperse and unaffected by the gel’s viscosity in the presence of other ingredients (surfactants and lipids) [44]. In combination with Carbopol, HPMC K4M is also used for the controlled release of drugs from nanocarriers and the stability of the formulation. In vitro diffusion of HPD-NLC gel illustrated higher release than free drug-loaded gel; however, nanocarriers-loaded gel showed a sustained release pattern. The results of release kinetics indicated that the Korsmeyer–Peppas model provided the best fit for the drug release kinetics from HPD-NLC-Gel. The drug release mechanism from HPD-NLC-Gel showed non-Fickian (anomalous) diffusion, as indicated by the release exponent “n” value, which was 0.506 (n < 0.45). This type of diffusion shows a complex type of release mechanism that interplays between swelling, gelation, and erosion due to a combination of polymers (i.e., HPMC and Carbopol) used in the formulation of gel. Earlier, the literature presented this kind of release mechanism especially when a combination of polymers is used in formulation [45].

In vivo studies were performed to evaluate the potential therapeutic effectiveness of loaded nano gel using imiquimod-induced psoriasis. This phenomenon of inducing psoriasis is established in preclinical settings, where imiquimod is widely used as a standard drug that activates the immune system via toll-like receptor 7 and thus pro-inflammatory cytokine cascade [39]. Furthermore, psoriatic skin typically displays keratosis, hyperkeratosis, parakeratosis, and sometimes orthokeratosis. Keratosis manifests as rough, reddish bumps on the skin. Hyperkeratosis involves the thickening of the stratum corneum due to excess keratin and often includes an enlarged granular layer. Parakeratosis, seen in conditions with increased cell turnover like psoriasis, is characterized by keratinization and thinning or loss of the granular layer. Orthokeratosis, by contrast, represents normal keratinization with the formation of a keratin layer devoid of nuclei, signifying a healthy epidermis. Pharmacodynamic evaluations highlighted a noticeable reduction in key pathological markers of psoriasis, including hyperkeratosis and parakeratosis, as observed in histological sections [46].

## 5. Conclusions

The current work exemplified the systemic QbD approach for the development and optimization of nanostructured lipid carriers loaded gel utilizing hesperidin as a drug for psoriasis treatment through a topical route of administration. The optimized formulation illustrated favorable physicochemical properties, sustained release of the drug, and improved psoriasis treatment in the preclinical imiquimod-induced disease model. The in vivo results were promising with the PASI score reductions and histopathological evaluations, all of which further demonstrated the clinical relevance for the treatment of psoriasis. Hence, it was concluded that HPD-NLC gel showed promising advancement in nanotechnology-based psoriasis management. The future direction of this research will be the scale-up of the project, conducting clinical trials to validate the safety and efficacy of the formulation.

## Figures and Tables

**Table 1:** The input parameters utilized during the BBD process to produce the formulations for HPD-NLCs.

Trial	Quantity of Drug (mg) (A)	Amount of Total Lipid (mg) (B)	Quantity of Liquid Lipid (mg) (C)	Surfactant Amount (% *w*/*v*) (D)
1	20	100	20	1
2	10	75	20	1
3	20	100	20	1
4	20	100	15	0.5
5	10	125	20	1
6	20	100	15	1.5
7	30	125	20	1
8	20	100	25	0.5
9	20	100	25	1.5
10	30	75	20	1
11	20	75	25	1
12	20	75	15	1
13	30	100	20	1.5
14	20	100	20	1
15	30	100	20	0.5
16	20	125	15	1
17	20	100	20	1
18	10	100	20	1.5
19	20	125	25	1
20	10	100	20	0.5
21	20	100	20	1
22	10	100	25	1
23	30	100	15	1
24	20	100	20	1
25	20	125	20	1.5
26	10	100	15	1
27	20	75	20	0.5
28	20	75	20	1.5
29	20	125	20	0.5
30	30	100	25	1

**Table 2:** The list of values of output parameters (particle size, PDI, and %EE) that resulted after the production of different trials of HPD-NLCs.

Trial	Particle Size (nm) (Y1)	Polydispersity Index (PDI) (Y2)	Entrapment Efficiency (%EE) (Y3)
1	202.4	0.239	40.21
2	206.5	0.267	35.81
3	209.4	0.271	38.29
4	154.2	0.246	22.28
5	237.8	0.264	39.54
6	224.5	0.265	51.82
7	166.3	0.261	26.22
8	350.7	0.371	30.85
9	176.1	0.234	51.5
10	204.6	0.338	41.43
11	180.4	0.285	25.62
12	136.8	0.264	35.08
13	169.8	0.314	44.27
14	204.3	0.261	38.54
15	137.6	0.251	32.81
16	139.4	0.287	32.25
17	149.5	0.269	29.52
18	169.4	0.274	38.11
19	198.7	0.365	28.54
20	128.5	0.244	29.52
21	195.4	0.314	40.12
22	172.5	0.291	38.06
23	135.8	0.243	52.26
24	139.5	0.261	48.24
25	155.0	0.275	38.95
26	125.9	0.248	44.57
27	142.8	0.262	34.28
28	136.4	0.277	51.25
29	161.8	0.284	41.08
30	180.2	0.289	32.43

**Table 3:** X-ray diffraction peaks of Hesperidin (HPD).

Pos. [°2θ]	FWHM Total [°2θ]	d-Spacing [Å]	Rel. Int. [%]	Area [cps*°2θ]
19.5482	0.2670	4.53746	23.49	13.25
21.4960	0.5660	4.13052	100.00	92.00
23.6328	1.0201	3.76166	38.47	63.91
35.9834	0.8759	2.49385	1.66	2.24
40.2200	0.8514	2.24039	2.00	1.83

**Table 4:** X-ray diffraction peaks of Compritol^®^ 888 ATO.

Pos. [°2θ]	FWHM Total [°2θ]	d-Spacing [Å]	Rel. Int. [%]	Area [cps*°2θ]
19.4499	0.5714	4.56019	11.91	3.40
21.3946	0.5660	4.14987	100.00	44.89
23.5587	1.2221	3.77333	38.25	24.78
28.2286	0.3897	3.16143	8.06	1.50

**Table 5:** X-ray diffraction peaks of physical mixture.

Pos. [°2θ]	FWHM Total [°2θ]	d-Spacing [Å]	Rel. Int. [%]	Area [cps*°2θ]
19.2804	0.5879	4.59989	13.21	7.97
21.2232	0.5656	4.18298	100.00	87.33
23.3769	0.8592	3.80225	38.17	53.88
35.8121	0.7793	2.50746	2.39	1.83

**Table 6:** X-ray diffraction peaks of Optimized HPD-NLC.

Pos. [°2θ]	FWHM Total [°2θ]	d-Spacing [Å]	Rel. Int. [%]	Area [cps*°2θ]
28.1451	0.3960	3.16800	100.00	2.24

**Table 7:** Composition of HPD-NLC loaded Gel.

Ingredients	HPD-NLC Loaded Gel
Carbopol 934	1.5%
HPMC K4M	0.5%
Glycerol	5 mL
Methyl Paraben	0.5%
Triethanolamine	2.5 mL
0.1 N NaOH	2.5 mL
Distilled water	q.s. *

* q.s.—quantity sufficient.

**Table 8:** The stability parameters under different storage conditions.

Storage Time (Months)	Storage Conditions					
	5 ± 3 °C		25 ± 2 °C/60 ± 5% RH		40 ± 2 °C/75 ± 5% RH	
	pH	Viscosity (cP)	pH	Viscosity (cP)	pH	Viscosity (cP)
0	6.5 ± 0.3	3546 ± 47	6.5 ± 0.3	3546 ± 47	6.5 ± 0.3	3546 ± 47
3	6.4 ± 0.5	3552 ± 35	6.4 ± 0.5	3470 ± 52	6.6 ± 0.5	3157 ± 56
6	6.5 ± 0.3	3513 ± 58	6.4 ± 0.5	3441 ± 34	6.3 ± 0.3	2889 ± 104
9	6.4 ± 0.5	3557 ± 96	6.3 ± 0.7	3447 ± 28	-	-
12	6.3 ± 0.3	3481 ± 35	6.4 ± 0.5	3385 ± 82	-	-

**Table 9:** Average PASI score for different groups.

Group	Erythema		Scaling		Thickness		Average PASI Score	
	Before	After	Before	After	Before	After	Before	After
I-Normal	0	0	0	0	0	0	0	0
II-Disease	2	2	3	3	2	2	2.3	2.3
III-Placebo gel	2	1	3	2	2	2	2.3	1.7
IV-HPD-Gel	2	2	3	1	2	2	2.3	1.3
V-Standard	2	1	3	1	2	1	2.3	1
VI-HPD-NLC-Gel	2	0	3	0	2	1	2.3	0.3

## Data Availability

The original contributions presented in this study are included in the article/Supplementary Materials. Further inquiries can be directed to the corresponding authors.

## Supplementary Materials

The following supporting information can be downloaded at: https://www.mdpi.com/article/10.3390/pharmaceutics17040478/s1, Tables S1 and S2: Data about the preliminary studies and pre-formulation assessments; Figures S1–S9: Results of preliminary studies and pre-formulation studies; Figure S10: Physical appearance of the formulated gel.

## Abbreviations

The following abbreviations are used in this manuscript:

BBD	Box–Behnken design
CCD	Central composite designs
CCP	Chitkara college of pharmacy
CCSEA	Committee for control and supervision of experiments on animals
DSC	Differential scanning calorimetry
EE	Entrapment efficiency
FTIR	Fourier transform infrared
g	grams
H&E	Hematoxylin and eosin
HPD	Hesperidin
HPMC	Hydroxypropyl methylcellulose
HRTEM	High-resolution transmission electron microscopy
IAEC	Institutional animal ethical committee
ICH	International council on harmonisation
NIPER	National institute of pharmaceutical education and research
NLCs	Nanostructured lipid carriers
OECD	Organisation for economic cooperation and development
PASI	Psoriatic area and severity index
PBS	Phosphate buffer saline
PDI	Polydispersity index
QbD	Quality by design
RH	Relative humidity
SLNs	Solid lipid nanoparticles
SLS	Sodium lauryl sulphate
XRD	X-ray diffraction
