# Supplementary material for: Nanostructured Lipid Carriers (NLC)-Based Topical Formulation of Hesperidin for Effective Treatment of Psoriasis
Source: Pharmaceutics. 2025 Apr 7;17(4):478. doi: 10.3390/pharmaceutics17040478 (PMC12030045; doi:10.3390/pharmaceutics17040478)
Supplement: Supplementary file 1 [file pharmaceutics-17-00478-s001.zip › pharmaceutics-3472068-supplementary.pdf]

Manuscript ID: pharmaceutics-3472068

Supplementary materials

Nanostructured lipid carriers (NLC)-based topical formulation of Hesperidin for effective treatment of psoriasis

Anita Rani[1], **Rajwinder Kaur**[1,*], **Afaf Aldahish**[2], **Rajalakshimi Vasudevan**[2], **Prasanalakshmi Balaji**[3,*], **Chander Parkash Dora**[1], **Balakumar Chandrasekaran**[4], **Thakur Gurjeet Singh**[1], and **Rahul Sharma**[5]

[1]Chitkara College of Pharmacy, Chitkara University, Rajpura 140401, Punjab, India
[2]Department of Pharmacology, College of Pharmacy, King Khalid University, Abha 61421, Saudi Arabia
[3]Department of Computer Science, College of Computer Science, King Khalid University, Abha 61421, Saudi Arabia.
[4]Faculty of Pharmacy, Philadelphia University, P. O. Box 1, Amman 19392, Jordan.
[5]Gateway College of Pharmacy, Sonepat 131001, Haryana, India.

*Corresponding authors: rajwinder.kaur@chitkara.edu.in (Rajwinder Kaur) and prengaraj@kku.edu.sa (Prasanalakshmi Balaji)

- **Preliminary study has been performed and the relevant data is collected in the supplementary Tables S1 & S2.**

Table S1. Summary of validation parameters of UV-spectroscopy for Hesperidin

**Parameter**	**Result**
λ_{max} (nm)	285
Regression equation (y= mx + c)	Y = 0.02301*X + 0.01073
Regression coefficient (r^2)	0.9981
Linearity (r^2)	0.9988, 0.9984 and 0.9989
**Accuracy (% mean drug recovery)**	
5 µg/ml	99.8%
10 µg/ml	101.24%
15 µg/ml	99.7%
20 µg/ml	98.76%
25 µg/ml	101.57%
30 µg/ml	101.32%
35 µg/ml	99.95%
40 µg/ml	100.43%
Intra-day precision indicated by % RSD for HPD	% RSD < 2%
Inter-day precision indicated by % RSD for HPD	% RSD < 2%
Intermediate precision indicated by % RSD for HPD	% RSD < 2%
Robustness indicated by % RSD	% RSD < 2%
Specificity (% mean recovery of drug)	100.46 %
Limit of detection (LOD)	1.29 µg/ml
Limit of quantitation (LOQ)	3.9 µg/ml

Table S2. Linear regression statistical data of calibration curve for HPD

Parameter	Value
**Best-fit values**	
**Slope**	0.02301 ± 0.0003772
**Y-intercept when X=0.0**	0.01073 ± 0.008979
**X-intercept when Y=0.0**	-0.4664
**1/slope**	43.45
**95% Confidence intervals**	
**Slope**	0.02212 to 0.02391
**Y-intercept when X=0.0**	-0.01050 to 0.03197
**X-intercept when Y=0.0**	-1.436 to 0.4420
**Goodness of fit**	
***R* square**	0.9981
***P* value**	< 0.0001
**Equation**	Y = 0.02301*X + 0.01073

➢ **Preliminary study has been performed, and the relevant results are presented as supplementary Figures S1 to S10.**

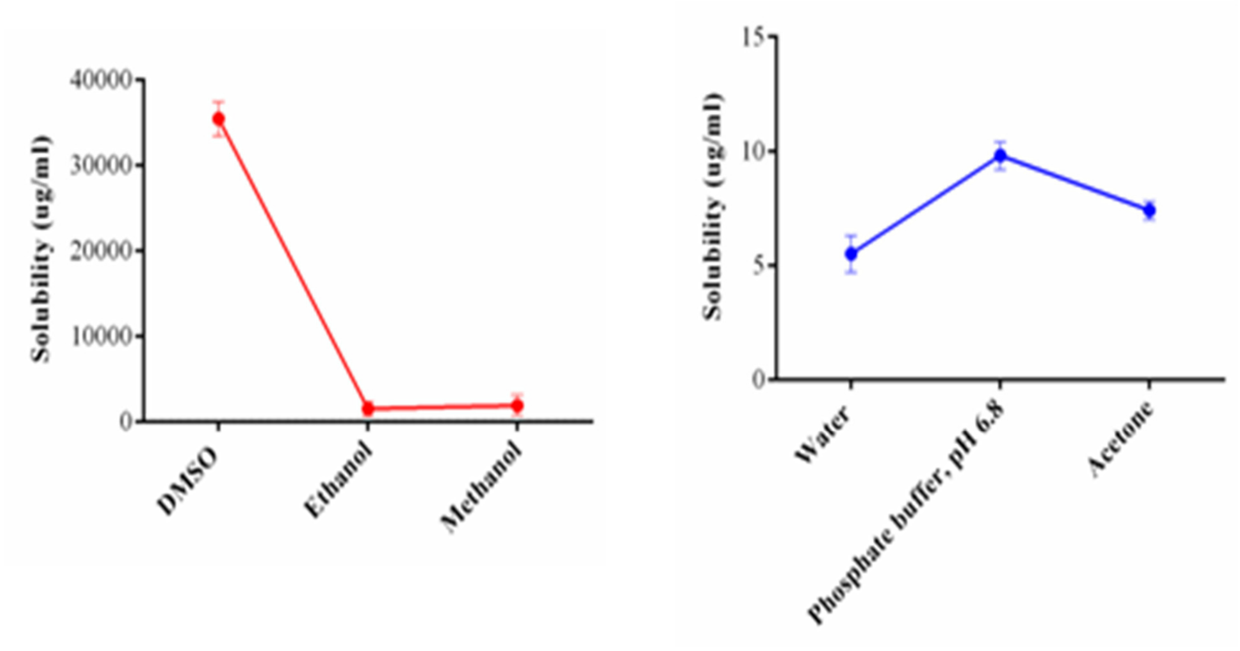

Figure S1. Solubility of Hesperidin in various solvents

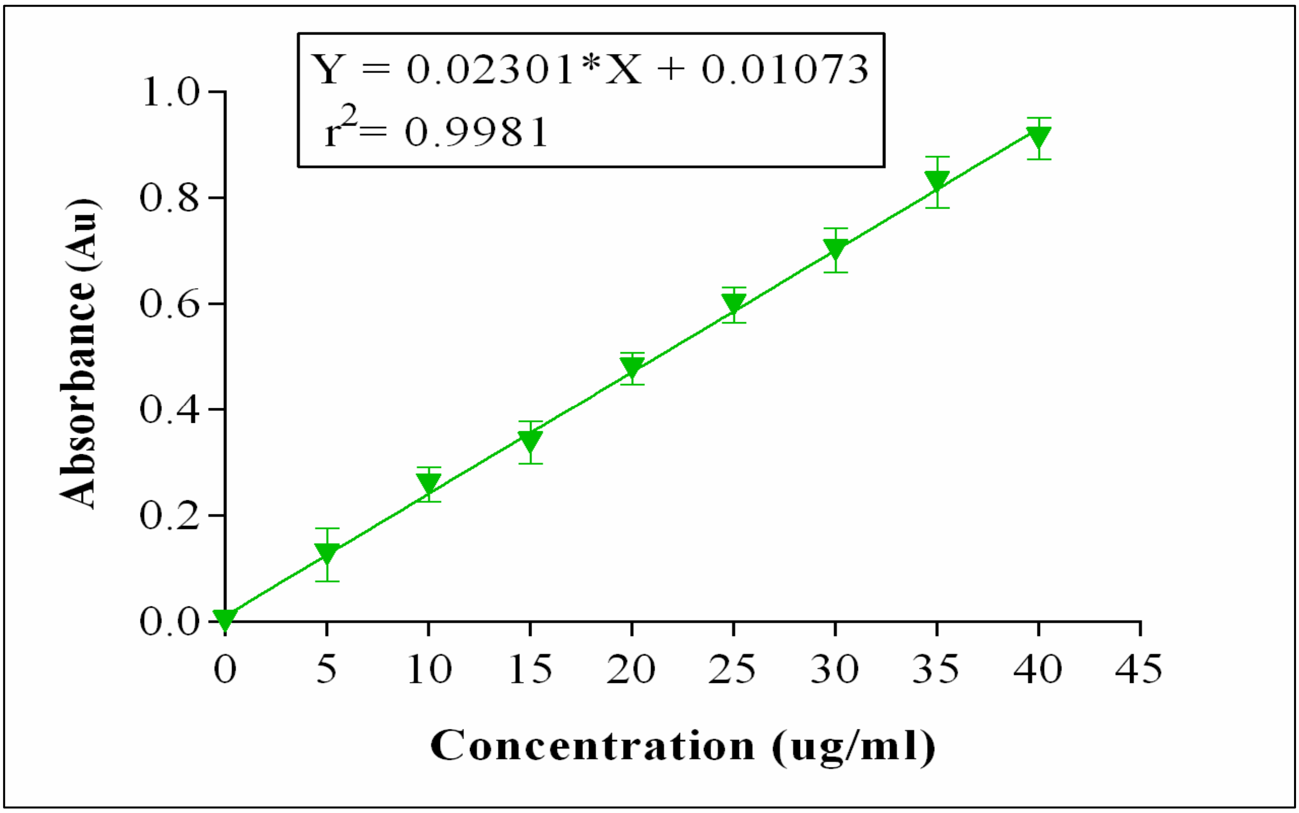

Figure S2. Standard curve of HPD in phosphate buffer, pH 6.8 using UV-spectroscopy

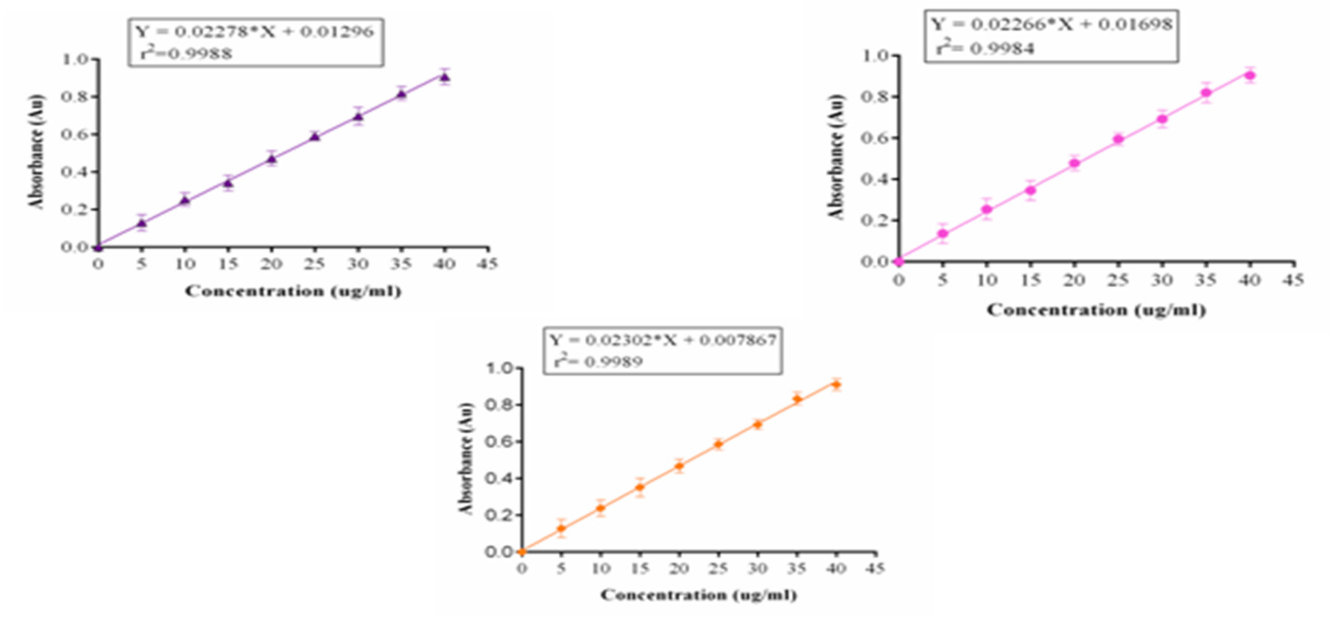

Figure S3. Linearity of UV-spectrophotometer analytical procedure for HPD (n=3)

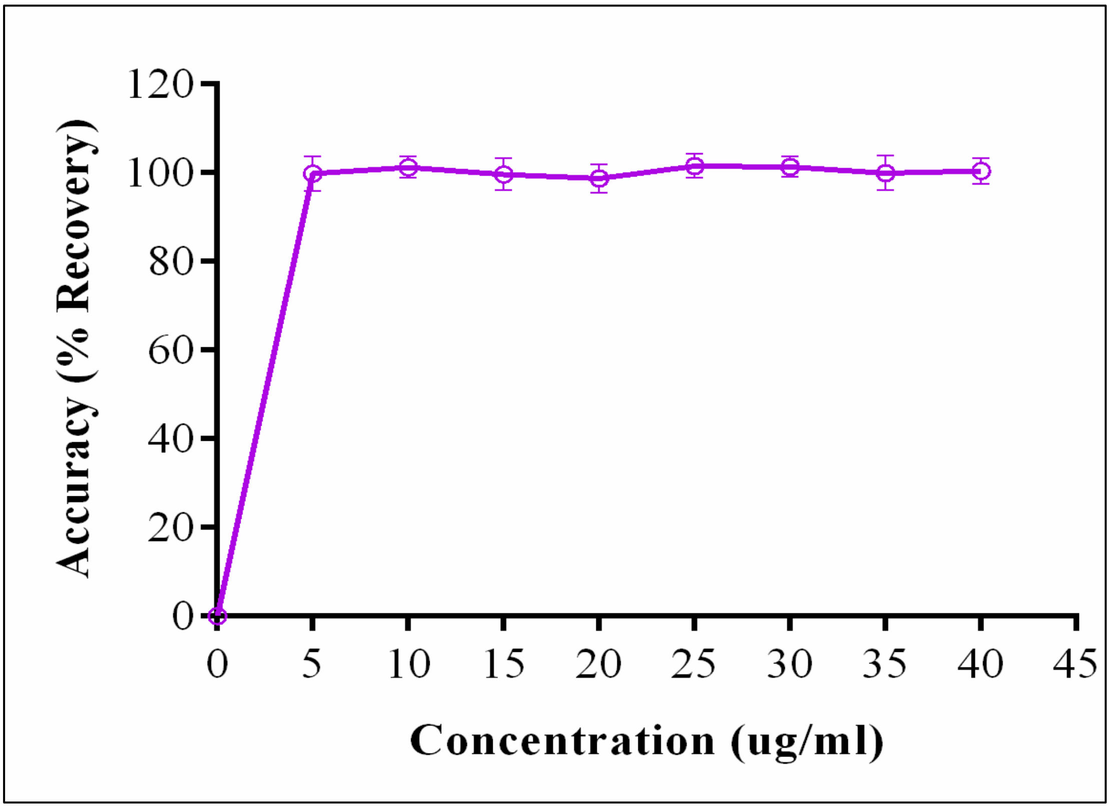

Figure S4. Accuracy of UV-spectrophotometer analytical method for HPD

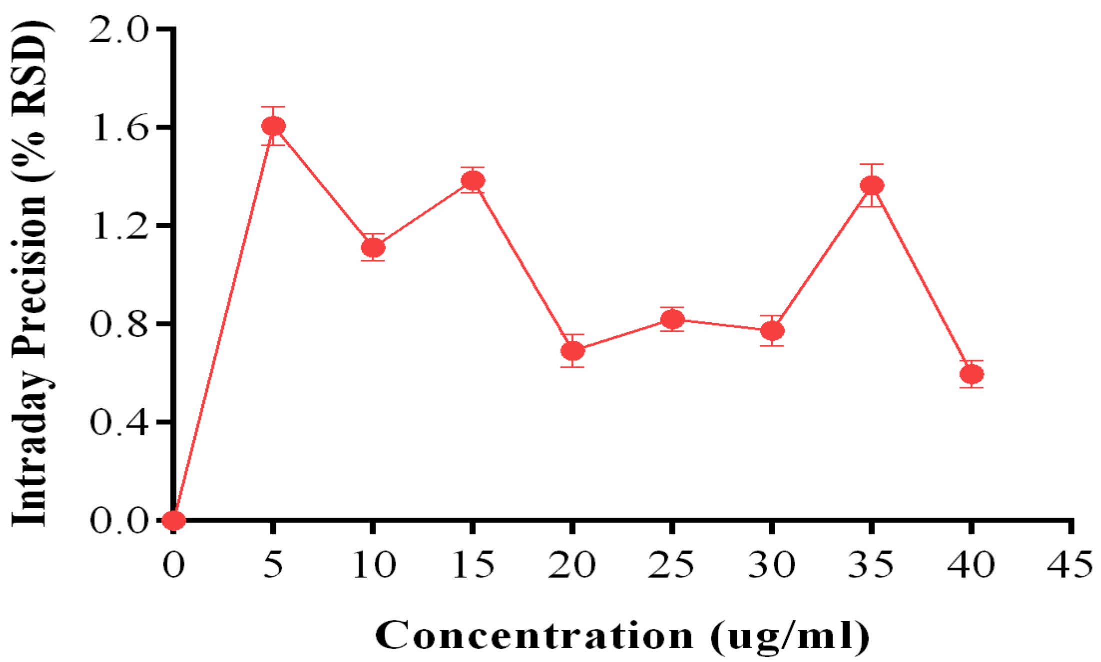

Figure S5. Intra-day precision determined for three different concentrations of HPD

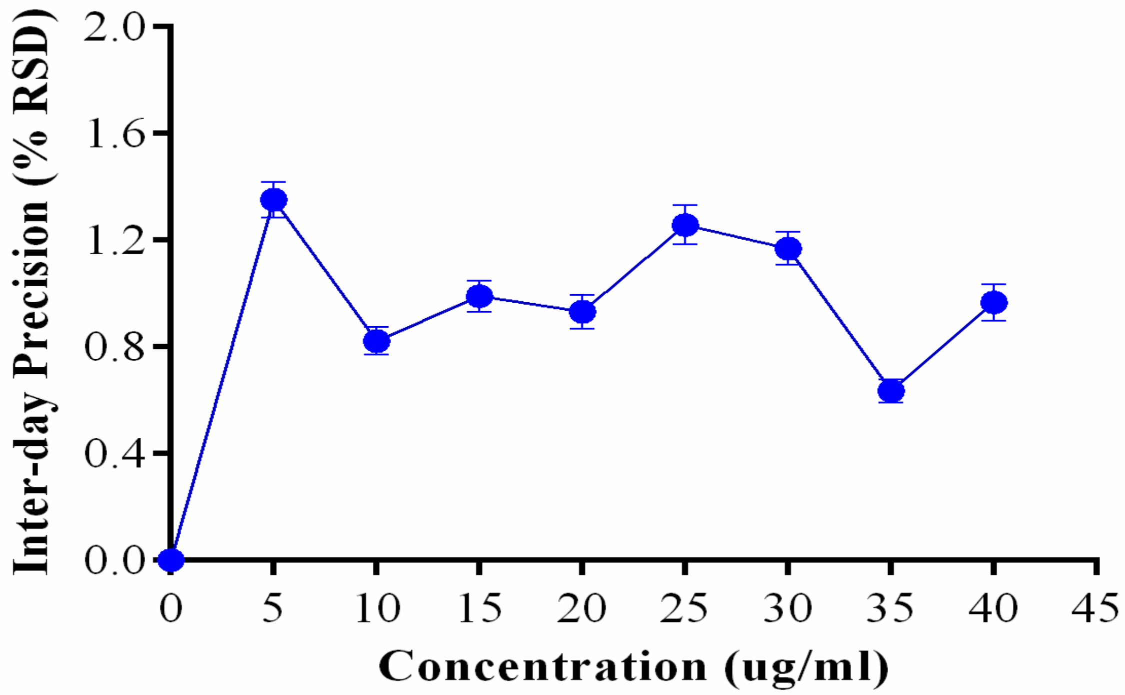

Figure S6. Inter-day precision determined for three different concentrations of HPD

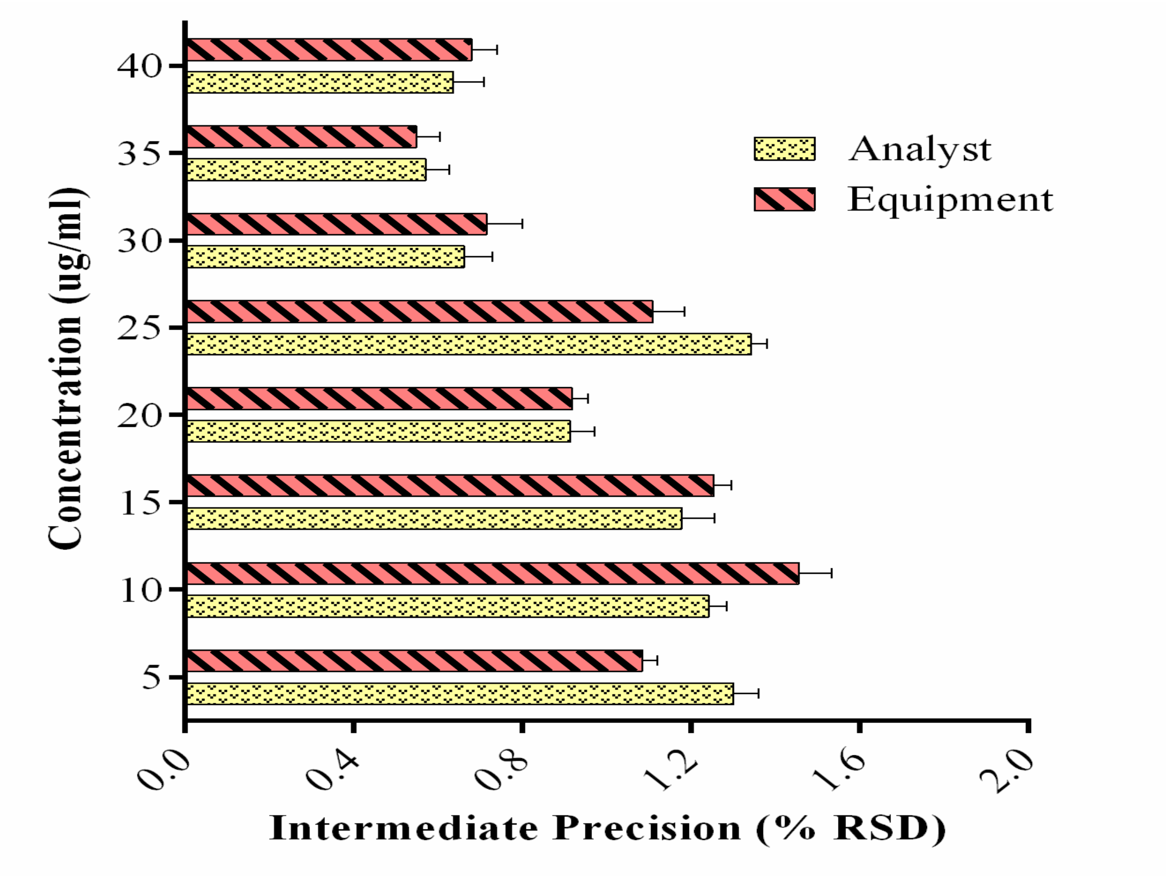

Figure S7. Intermediate precision of the analytical method for HPD (n = 8).

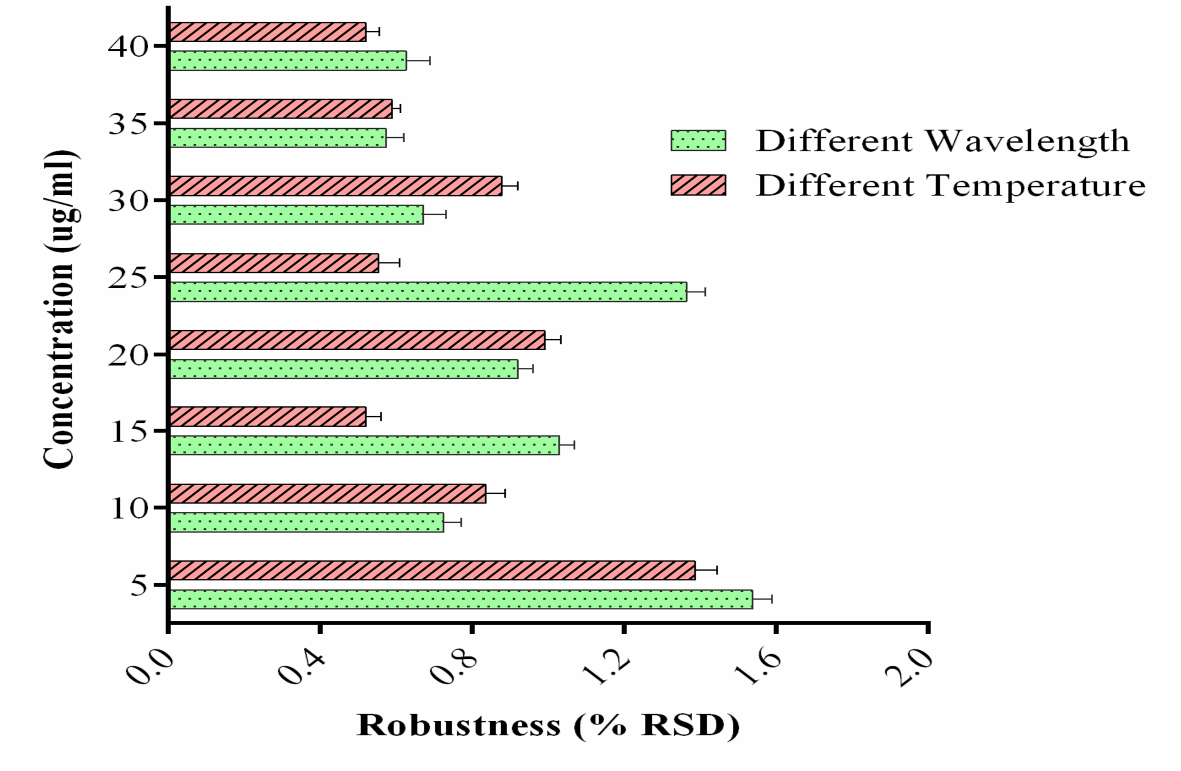

Figure S8. Robustness of analytical method for HPD (n = 8)

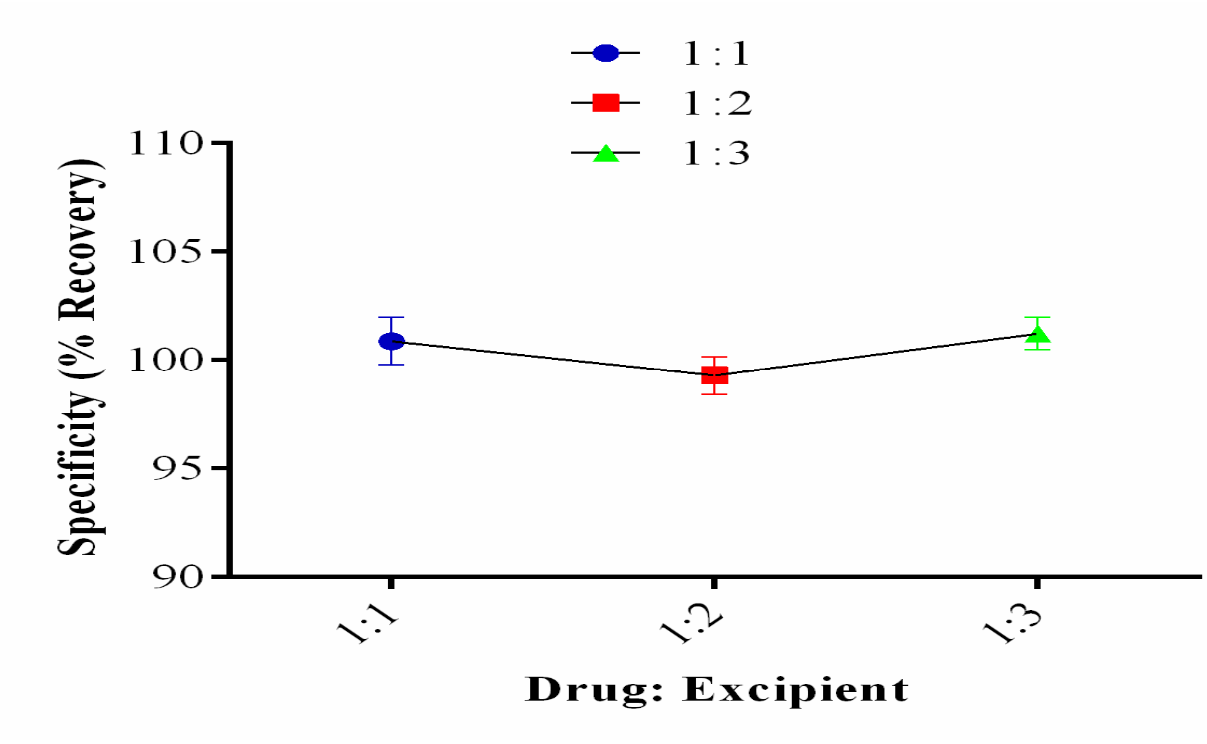

Figure S9. Specificity of analytical method for HPD

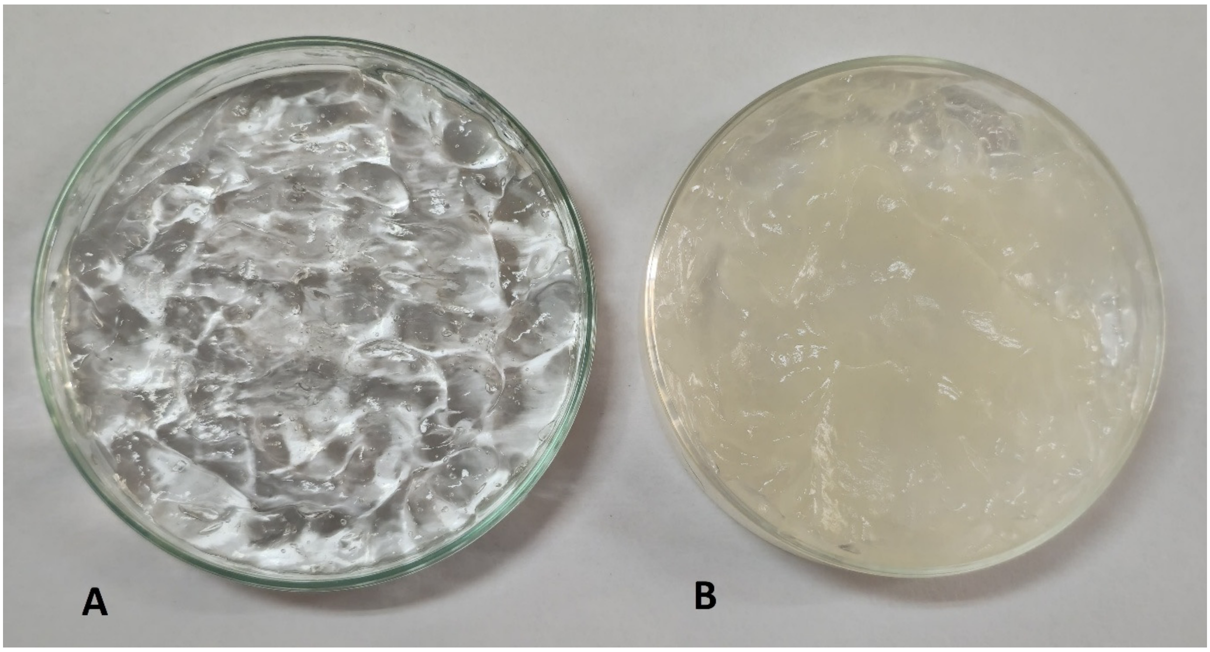

Figure S10. The physical appearance of (**A**) plain gel and (**B**) HPD-NLC gel

----
